# Characterization and Determination of 2-(2-Phenylethyl)chromones in Agarwood by GC-MS

**DOI:** 10.3390/molecules181012324

**Published:** 2013-10-08

**Authors:** Wen-Li Mei, De-Lan Yang, Hao Wang, Jin-Ling Yang, Yan-Bo Zeng, Zhi-Kai Guo, Wen-Hua Dong, Wei Li, Hao-Fu Dai

**Affiliations:** 1 Key Laboratory of Biology and Genetic Resources of Tropical Crops, Ministry of Agriculture, Institute of Tropical Bioscience and Biotechnology, Chinese Academy of Tropical Agricultural Sciences, Haikou 571101, China; 2 Hainan Key Laboratory for Research and Development of Natural Product from Li Folk Medicine, Haikou 571101, China

**Keywords:** agarwood, 2-(2-phenylethyl)chromone, fragmentation pattern, GC-MS

## Abstract

Agarwood is the fragrant resinous heartwood obtained from certain trees in the genus *Aquilaria* belonging to the family Thymelaeaceae. 2-(2-Phenylethyl)chromones and characteristic sesquiterpenes are the main classes of aromatic compounds isolated from agarwood. Although there are many sesquiterpenes, relatively few 2-(2-phenylethyl)chromones have been determined in agarwood by GC-MS. After analysis of the MS spectra of eighteen 2-(2-phenylethyl)chromone derivatives isolated from agarwood and identified by NMR spectroscopy, together with the reported MS data and characteristic of structures of 2-(2-phenylethyl)chromones, the MS characterization, fragmentation patterns and characteristic fragment peaks for the compounds were deduced and a table summarizing MS characterization of 2-(2-phenylethyl)chromones in agarwood is presented. All the 2-(2-phenylethyl)chromones previously reported in agarwood are substituted by methoxy or/and hydroxy groups, except for one compound. Due to the fact they all possess the same basic skeleton (molecular weight: 250) and similar substituent groups (methoxy or hydroxy groups), a formula (30m + 16n = MW − 250) is provided to calculate the number of methoxy (m) or hydroxy (n) groups according to molecular ion peak or molecular weight (MW). We deduced that the characteristic fragmentation behaviors of the 2-(2-phenylethyl)chromones are the cleavages of the CH_2_-CH_2_ bond between chromone moiety and phenyl moiety. Thus, characteristic fragment ions, such as *m*/*z* 91 [C_7_H_7_], 107 [C_7_H_6_+OH], 121 [C_7_H_6_+OCH_3_], 137 [C_7_H_5_+OH+OCH_3_] are formed by different substituted benzyl moieties, while characteristic fragment ions such as *m*/*z* 160 [C_10_H_8_O_2_], 176 [C_10_H_7_O_2_+OH], 190 [C_10_H_7_O_2_+OCH_3_], 220 [C_10_H_6_O_2_+OCH_3_×2] are formed by different substituted chromone moieties. Furthermore, rules regarding to the relationship between the positions of hydroxy or methoxy groups and the relative abundances of benzyl and chromone fragment ions have been deduced. Elucidation of how the positions of hydroxy or methoxy groups affect the relative abundances of benzyl and chromone fragment peaks is also provided. Fifteen unidentified compounds of an artificial agarwood sample analyzed by GC-MS, were preliminary determined as 2-(2-phenylethyl)chromones by analysis of their MS characterization and by comparison of their MS spectra with those of 18 standard compounds or 2-(2-phenylethyl)chromones reported in literature according to the above-mentioned methods and rules. This report will be helpful for the analysis and structural elucidation of 2-(2-phenylethyl)chromones in agarwood by GC-MS, and provides fast and reliable characterization of the quality of agarwood.

## 1. Introduction

Agarwood is a fragrant resinous wood obtained from certain trees in the genus *Aquilaria* belonging to the family Thymelaeaceae [1,2]. The precious, high-priced, fragrant agarwood, also called chen-xiang in Chinese, gaharu and kalambak in Malaysia, kanankoh and jinkoh in Japan, oudh, eaglewood, agar, ghara, and aloeswood, has been used for centuries as incense in Buddhist, Hindu and Islamic ceremonies [1,2]. It also plays an important role in Chinese Traditional Medicine for use as a sedative and carminative, and to relieve gastric problems, coughs, rheumatism and high fever [3]. The healthy wood of *Aquilaria* trees is white, soft and without scented resins. It is widely accepted that the dark resinous material of *Aquilaria* is created as a response to some form of injury to the tree, including natural injuries, such as lightning strikes, animal grazing, insect attacks or microbial invasions, and artificial injuries, such as cutting, nailing, holing, fire, chemical wounding, and deliberate fungi inoculation [2,4,5,6]. Agarwood formation occurs slowly and infrequently in Nature and the supply of agarwood from wild sources is far less than market demand. Because of its immense value and rarity, indiscriminate cutting of trees and overharvesting in hope of finding the treasured resin has led to the depletion of wild trees [7,8]. Nine *Aquilaria* species, including *A. sinensis*, were listed on the IUCN red list as endangered species [9]. Since 2004 all species of *Aquilaria* have been placed on the Appendix II list of the Convention on International Trade in Endangered Species of Wild Fauna and Flora [1]. Trade in agarwood has intensified in recent years due to demand and commercial value. Prices for agarwood products range greatly from US$100/kg up to US$100,000/kg for different qualities [1]. All sorts of false agarwood have emerged in the market, so the establishment of scientific and reliable methods to evaluate agarwood quality is very important.

Chemical analyses of agarwood show that its most abundant components are 2-(2-phenylethyl)-chromone derivatives (41%) and other sesquiterpenes (52%) [2]. 2-(2-Phenylethyl)chromone derivatives of agarwood have been isolated and identified by our previous work [10,11,12,13]. Despite the occurrence of many sesquiterpenes, only a few 2-(2-phenylethyl)chromones have been determined by GC-MS for agarwood [14,15,16,17]. This paper summarizes MS characterization of 2-(2-phenylethyl)chromones and provides methods to characterize 2-(2-phenylethyl)chromones in agarwood by analysis of their characteristic MS fragmentation patterns and by comparison of their MS spectra with reported ones. This report should be helpful for the analysis and structural elucidation of 2-(2-phenylethyl)chromones in agarwood by GC-MS, and provides a scientific basis for the fast, simple, and sound evaluation of the quality of agarwood.

## 2. Results and Discussion

**Figure 1:**
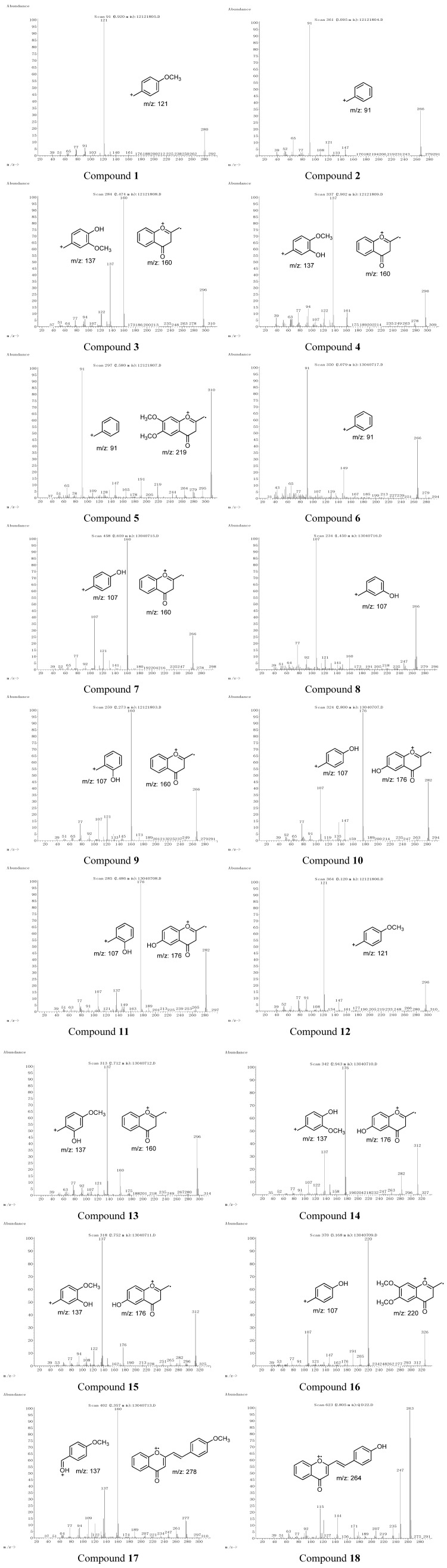
MS spectra of compounds **1**–**18** and the structures of characteristic fragment ions.

2-(2-Phenylethyl)chromone derivatives **1**–**18** were isolated from Chinese agarwood in our laboratory, and their structures identified by NMR analysis [10,11,12,13]. By comparison of the MS spectra of 2-(2-phenylethyl)chromones **1**–**18** (Figure 1), it was determined that these chromones have very similar characteristic mass spectra. A molecular ion peak (in the relative molecular mass range from 250 to 400, the abundance is usually under 60%), together with a fragment base peak, and sometimes also one or two another fragment peaks (abundance of more than 30%) can be observed, while the abundances of other fragment peaks are mostly less than 10%. The fragment base peak and the main fragment peaks were mostly at *m*/*z* 91, 121, 137, 107, 160, 176, 190, 220, *etc*.

**Scheme 1:**
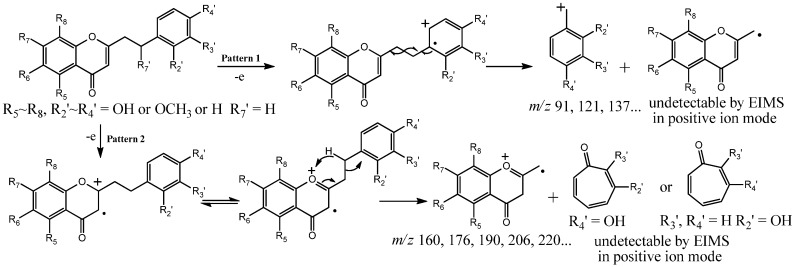
Proposed MS fragmentation patterns and characteristic fragments of 2-(2-phenylethyl)chromone.

By analyzing the molecular ion peaks and the characteristic fragment ion peaks of compounds **1**–**18**, we deduce that the CH_2_-CH_2_ bond which connects the chromone moiety with the phenyl moiety was cleaved after electron impact (Scheme 1). Thus, the hydroxyl- or methoxy-substituted benzyl moieties provide characteristic ions at *m*/*z* 91 [C_7_H_7_], 107 [C_7_H_6_+OH], 121 [C_7_H_6_+OCH_3_], 137 [C_7_H_5_+OH+OCH_3_], *etc.*; while the hydroxyl- or methoxy-substituted chromone moieties provide characteristic ions at *m*/*z* 160 [C_10_H_8_O_2_], 176 [C_10_H_7_O_2_+OH], 190 [C_10_H_7_O_2_+OCH_3_], 220 [C_10_H_6_O_2_+OCH_3_×2], *etc*.

To further verify the above conjecture, we analyzed all the 2-(2-phenylethyl)chromones isolated from agarwood and their MS data, which are listed in Table 1. Except for one compound (No. 32) substituted by acetoxyl group, all the other compounds were found to be substituted by hydroxy or/and methoxy groups. Thus, due to the fact all the 2-(2-phenylethyl)chromones isolated from agarwood share the same basic skeleton (molecular weight: 250) and similar substituent groups (mainly hydroxy and methoxy), the number of methoxy or hydroxy groups can be determined from the molecular ion peak, or molecular weight (MW) through the Formula (1), in which “m” means the number of methoxy groups, and “n” means the number of hydroxyl groups .


30m + 16n = MW − 250
(1)

After further analyzing the MS data in Table 1, some rules have been discovered which should be helpful for characterization of 2-(2-phenylethyl)chromones by MS data. The 6, 4′ positions are most likely to be substituted, closely followed by 7 and 3′, meanwhile, only three compounds, Nos. 33~35, were substituted at the position of 7′ and all by hydroxyl groups. Thus, first we analyze the 7′ non-substituted compounds Nos. 1~32, whose relative fragment abundances are affected by their substitution patterns. We found that usually the substitution of a benzyl moiety has a greater impact than the substitution of chromone moiety, and the effect of methoxy substitution is similar to that of no substitution, while the opposite is true of hydroxy substitution; the *p-*substituted (4′) groups have a greater impact than other positions on the relative abundance of fragments. Detailed analysis is as follows:

(1) (a) the benzyl moiety is non-substituted; (b) the benzyl moiety is substituted only by methoxy; (c) the benzyl moiety is substituted by both methoxy and hydroxy, as well as methoxy in the *p-*position (position 4′). All of the above mentioned situations favor the formation of benzyl fragment ions (*m/z* 91, 121, 137), and in most cases these fragment ions are the base peaks;
(2) The benzyl moiety is substituted by hydroxy: (a) *p*- or *o*-hydroxy substitution favors the formation of chromone fragment peaks (*m*/*z* 160, 176, 190, 220); (b) *m-*hydroxy can increase the relative abundances of benzyl fragment ions (*m*/*z* 107, 137).

Meanwhile, the chromone moiety substitution has a weaker impact to the relative abundances of fragment ions, but the effects are not so critical and the rule is not so clear.

Thus, the distribution of substituent groups (−OH or −OCH_3_) at benzyl moiety and chromone moiety can be determined by the relative abundances of benzyl and chromone fragment peaks in MS spectra according to the above-mentioned rules.

The MS data in Table 1 will be taken for example to explain how to apply the rules. All the base peak of compounds Nos. 1~4, 8, 10, 11, 14, 19 are *m/z* 91, while the abundances of chromone fragment ions are very low or even negligible, corresponding to their non-substituted benzyl moiety as per rule 1(a). All the base peaks of compounds Nos. 9, 15, 20~23, 29 are *m/z* 121, while the abundances of chromone fragment ions are very low or even negligible, corresponding to their benzyl moiety substituted only by methoxy according to rule1(b).

**Table 1:** MS characterization of 2-(2-phenylethyl)chromones in agarwood.

No.	Chromone Moiety (CM)				Benzyl Moiety (BM)				M	RA (%)	BM	RA (%)	CM	RA (%)	R or NC
	R_5_	R_6_	R_7_	R_8_	R_4'_	R_3'_	R_2'_	R_7'_							
1									250	20	91	100			[18]
2		OH							266	36	91	100			**2**, [10]
3			OH						266	49	91	100			[19]
4				OH					266	58	91	100			**6**, [20]
5					OH				266	40	107	28	160	100	**7**,[10]
6						OH			266	100	107	58	160	10	**8**, [10]
7							OH		266	46	107	18	160	100	**9**, [10]
8		OCH_3_							280	48	91	100	161	10	[18,21]
9					OCH_3_				280	27	121	100	160	3	**1**, [22]
10	OH			OH					282	100	91	100	191	2	[23]
11		OH		OH					282	18	91	100	176	2	[19]
12		OH			OH				282	58	107	60	176	100	**10**, [19]
13		OH					OH		282	60	107	13	176	100	**11**, [19]
14		OH	OCH_3_						296	61	91	100	190	4	[19]
15		OH			OCH_3_				296	22	121	100			**12**, [18]
16					OH	OCH_3_			296	37	137	52	160	100	**3**, [10]
17					OCH_3_	OH			296	28	137	100	161	12	**4**, [10]
18					OCH_3_		OH		296	48	137	100	160	27	**13**, [10]
19		OCH_3_	OCH_3_						310	84	91	100	219	10	**5**, [18,21]
20		OCH_3_				OCH_3_			310	60	121	100	190	8	[18]
21		OCH_3_			OCH_3_				310	18	121	100	190	3	[22]
22		OH	OH		OCH_3_				312	12	121	100	191	8	[24]
23	OH			OH	OCH_3_				312	24	121	100			[23]
24		OH			OH	OCH_3_			312	38	137	33	176	100	**14**, [10]
25		OH			OCH_3_	OH			312	42	137	100	176	16	**15**, [25]
26		OCH_3_	OCH_3_		OH				326	41	107	23	220	100	16 *
27		OCH_3_			OH	OCH_3_			326	17	137	30	190	100	[19]
28		OH		OH	OH	OCH_3_			328	20	137	34	190	100	[26]
29		OCH_3_	OCH_3_		OCH_3_				340 (310)	18 (16)	121	100	220	2	[23]
30		OH	OCH_3_		OCH_3_	OH			342	60	137	100	206	72	[24]
31		OCH_3_	OCH_3_		OCH_3_	OH			356	75	137	81	220	100	[24]
32	OCH_3_			OCH_3_		OCOCH_3_			368	5	149	100			[21]
33					OCH_3_			OH	296 (277)	− (20)	137	43	160	100	17 *
34					OH			OH	282 (263)	− (100)					18 *
35		OH						OH	282 (263)	− (+)	107	100	176	+	[27]
36	OH	OCH_3_							296	+	91	100	190	+	[27]
37		OH	OCH_3_		OH				312	+	107	+	206	100	[24]
38		OCH_3_	OH		OCH_3_				326	+	121	100	206	+	[28]
39		OH		OH	OCH_3_	OH			328	+	137	100	192	+	[24]
40		OCH_3_	OH		OCH_3_	OH			342	+	137	100	206	+	[24]
41		OCH_3_	OCH_3_		OH	OCH_3_			356	+	137	+	220	100	[24]

RA: relative abundance; +: RA higher than 10%; －: RA less than 10%; *: new compound; R: reference; NC: Number of compounds **1**~**18**.

All the benzyl moieties of compounds Nos. 17, 18, 24, 30, 31 are substituted by a methoxy and a hydroxy, and a methoxy in the *p-*position (position 4′), in accordance with rule 1(c). Their base peak is *m/z* 137, except for compound No. 31, while the chromone fragment peaks showed different abundances for their different substituted chromone moieties. Taking compounds Nos. 30 and 31 as examples, both of them have the same *p*-methoxy- and *m-*hydroxy-substituted benzyl moiety, which favors the formation of a benzyl fragment ion at *m/z* 137. The chromone moiety of compound No. 30 is substituted by *p*-hydroxy and *m*-methoxy, which results in a benzyl base peak at *m/z* 137 and a 72% chromone fragment peak at *m/z* 206. However, the chromone moiety of compound No. 31 is substituted by both *m*- and *p*-methoxy, which results in the chromone base peak at *m/z* 220 and an 81% benzyl fragment peak at *m/z* 137.

The benzyl moiety of compounds Nos. 5, 7, 12, 13, 16, 25~28 are substituted by *p-* or *o*-hydroxy which are in favor of the formation of chromone fragment peaks (*m/z* 160, 176, 190, 220) in accordance with rule 2(a), while the benzyl fragment peaks showed different abundances for their different substituted chromone moieties. Taking compounds Nos. 5, 12, and 26 for example, all of the benzyl moieties are substituted by *p*-hydroxy groups, which favors the formation of chromone fragment peaks (*m/z* 160, 176, 220) as base peaks. However, due to the different substitution on the chromone moiety, the abundance of the benzyl fragment peak at *m/z* 107 of compound No. 12 is over 60%, while the abundance of the benzyl fragment peak at *m/z* 107 of compounds No. 5 and No. 26 are 28% and 23%, respectively.

The spectra were recorded with electron impact (EI) MS in positive ion mode, thus only cation ions, but no radical ions or neutral fragments can be detected. Based on the above results, we conjectured that the benzyl fragment cation ions and chromone fragment cation ions are most likely to be formed from two different cleavage methods. One is homolytic cleavage of the CH_2_-CH_2_ bond caused by radicals, which results in formation of benzyl fragment cation ions, such as *m*/*z* 91,121, 137,107, *etc*. (Scheme 1). Another is rearrangement fragmentation, which results in formation of chromone fragment cation ions, such as *m*/*z* 160, 176, 190, 220, *etc*. (Scheme 1). The occurrence of these two competitive cleavage mechanisms is mainly affected by the distribution of substituent groups (−OH or −OCH_3_) on the benzyl and chromone moieties. Under the conditions of rules 1(a)~1(c) and 2(b), the first cleavage method occurs primarily, which results in formation of benzyl fragment cation ions, while under the conditions of 2(a), the second cleavage method occurs primarily, which results in formation of chromone fragment cation ions.

The fragmentation patterns of 7′-hydroxy-substituted compounds, for example, compounds **17** and **18 ** (Nos. 33 and 34) , are discussed as follows: the dehydration fragment ion at *m*/*z* [M-18] was easily formed by chemical dehydration at the 7′ position, therefore, the abundances of molecular ions are very low or even non-existent. If the ethyl bond is broken before dehydration, the characteristic benzyl fragment or chromone fragment ions can be formed, such as the case of compound **17**, which shows the chromone base peak at *m/z* 160 and a 43% benzyl fragment ion at *m/z* 137. On the contrary, the whole molecule becomes a stable conjugated system after the vinyl is formed by dehydration, then it is difficult to break the vinyl bond, but various fragment peaks can be formed by dehydration or decarbonylation, such as in the case of compound **18** (Figure 1).

New 2-(2-phenylethyl)chromones have been reported, and some of these reports did not give any mass spectral data. The characteristic fragment peaks of these compounds (Nos. 35~41) were predicted on the base of the rules summarized above, and need to be verified experimentally in the future. Taking compound No. 41 for example, the substituent groups on the benzyl moiety, *p*-hydroxy and *m*-methoxy, are the same as in compound No. 27. Of them, the *p*-hydroxy favors the formation of a chromone fragment ion according to rule 2(a), while the *m*-methoxy favors the formation of a benzyl fragment ion according to rule 1(b). The chromone moiety of compound No. 41 is substituted by both *m*- and *p*-methoxy groups, similar to the only *p*-methoxy-substituted chromone moiety of compound No. 27. Finally, we deduce that the aggregated effect of the substituents on compound No. 41 results in the chromone base peak at *m/z* 220, while the benzyl fragment peak at *m/z* 137 and molecular ion peak at *m/z* 356 also appear, similar to compound No. 27.

It is worthwhile to note that the mass spectral data for 5,6,7,8-tetrahydro-2-(2-phenylethyl)chromones and diepoxytetrahydro-2-(2-phenylethyl)chromones identified in agarwood is not within the scope of the discussion in this paper.

**Figure 2:**
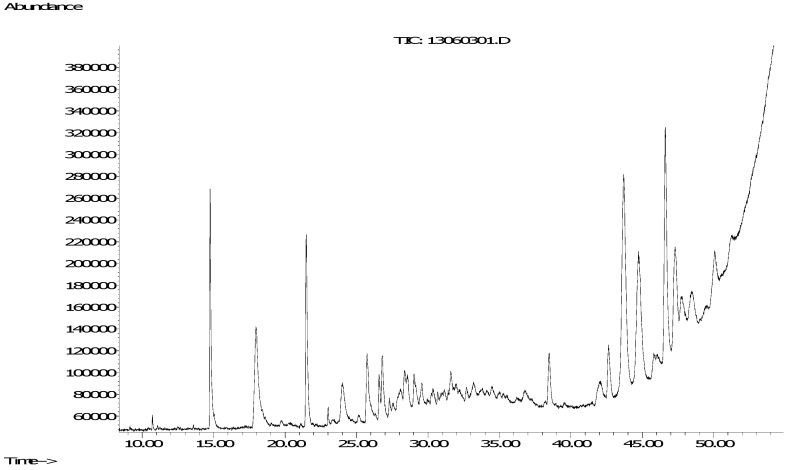
Total ion chromatogram of the agarwood oil.

Based on the abovementioned methods and rules, we analyzed an agarwood sample, which was harvested about nine months after injection of agarwood inducer into the trunk of a six-year-old *A. sinensis* tree at an *A. sinensis* plantation. At first, 0.5 mg of the agarwood oil was analyzed by the GC/MS method. Figure 2 shows the total ion chromatogram, and the compounds characterized according to matches with compounds in the NIST05 and WILEY275 databases are listed in Table 2. 

The results showed that only several simple volatile aromatic compounds, and an aliphatic acid, palmitic acid, together with 2-(2-phenylethyl)chromone have been identified, while in the retention times from 25.74 min to 38.59 min, and from 38.59 min to 51.24 min, there were a lot of compounds that were not identified. As we know, 2-(2-phenylethyl)chromone derivatives and characteristic sesquiterpenes are the main two compounds classes isolated from agarwood [1]. In addition, the molecular weights of sesquiterpenes are usually under 250, while the molecular weights of 2-(2-phenylethyl)chromones are at least 250. By comparing the retention times of the two kinds of compounds, we found that sesquiterpenes usually appear in front of 2-(2-phenylethyl)chromones. This rule can be found in the GC-MS analysis of agarwood of Japanese scholars and our group [14,15,16], so we deduced that the unidentified compounds with retention times from 25.74 min to 38.49 min are mainly sesquiterpenes, while the unidentified compounds with retention times from 38.49 min to 51.24 min are mainly 2-(2-phenylethyl)chromones, which was confirmed by their MS spectra.

**Table 2:** Chemical components and their relative content of agarwood oil.

No.	Retention time	Compound	Molecular formula	Molecular weight	Relative content (%)
YF1	10.72	Nonanal	C_9_H_18_O	142	0.19
YF2	14.76	Benzylacetone	C_10_H_12_O	148	5.18
YF3	17.96	Benzenepropanoic acid	C_9_H_10_O_2_	150	6.81
YF4	21.48	Anisylacetone	C_11_H_14_O_2_	178	5.94
YF5	24.02	3-(4-Methoxyphenyl)propionic acid	C_11_H_16_O_2_	180	2.59
YF6	25.74	Zingerone	C_11_H_14_O_3_	194	3.58
YF7	26.80	unidentified		206	2.57
YF8	31.59	Palmitic acid	C_16_H_32_O_2_	256	0.80
YF9	38.49	2-(2-phenylethyl)chromone	C_17_H_14_O_2_	250	2.07
YF10	42.03	unidentified		266	2.91
YF11	42.64	unidentified		280	2.82
YF12	43.71	unidentified		282	13.99
YF13	44.74	unidentified		266	11.22
YF14	45.81	unidentified		296	1.44
YF15	46.62	unidentified		310	9.84
YF16	47.30	unidentified		312	5.37
YF17	47.77	unidentified		296	3.14
YF18	48.46	unidentified		296	3.68
YF19	50.05	unidentified		328	3.44
YF20	51.24	unidentified		326	1.70

**Figure 3:**
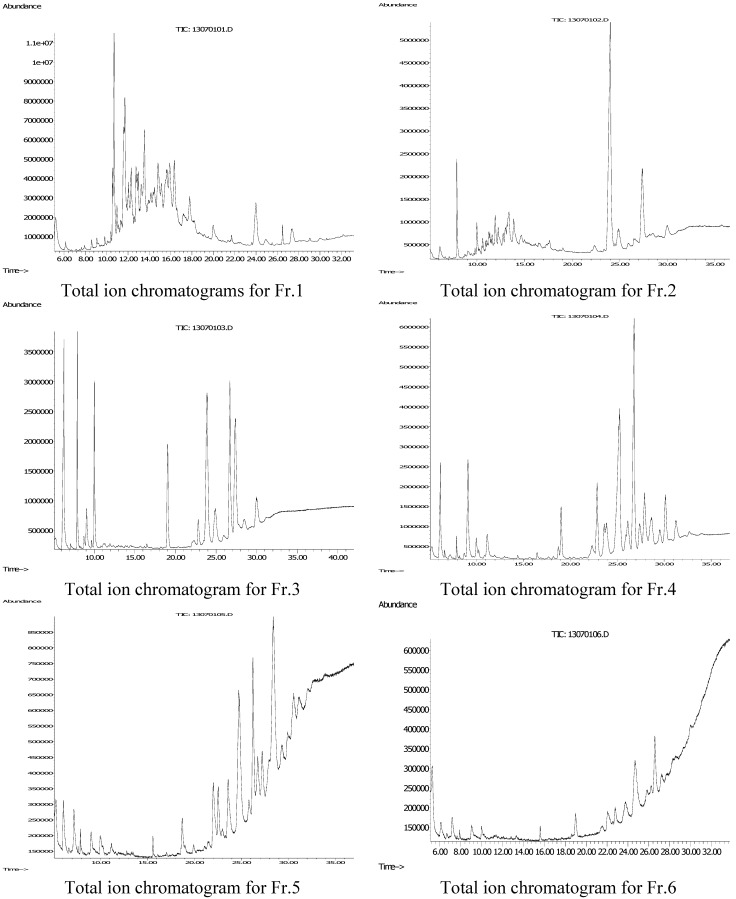
Total ion chromatogram for Fr.1~6 of agarwood oil.

**Figure 4:**
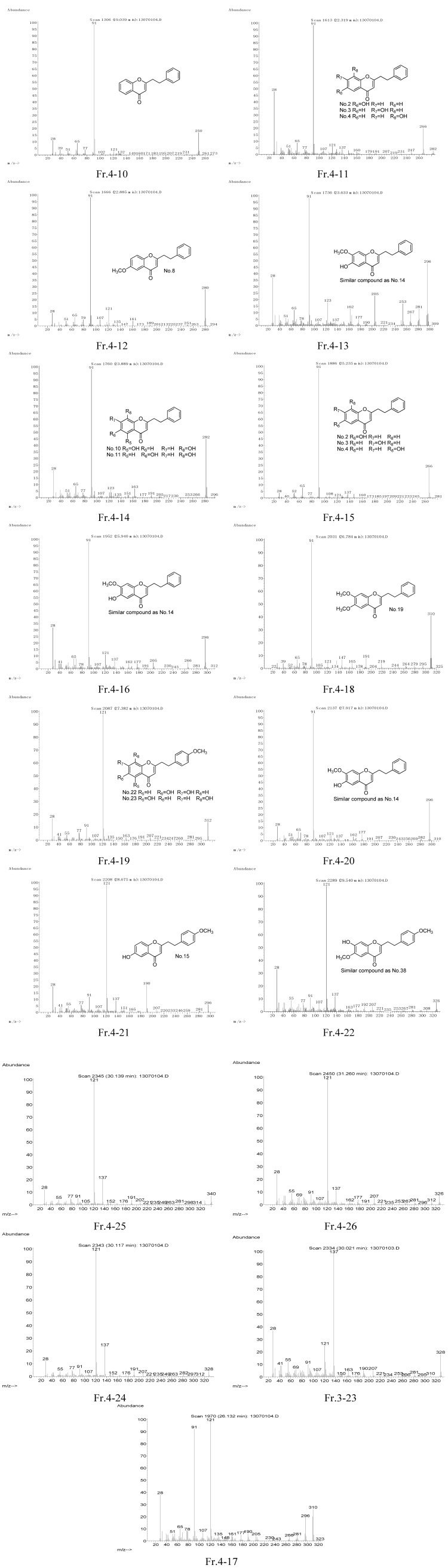
MS spectra and structures characterization of 2-(2-phenylethyl)chromones of fraction 4 of agarwood oil.

In order to focus on the determination of 2-(2-phenylethyl)chromones, 70 mg of the agarwood oil was dissolved in 0.5 mL MeOH, and then applied to a Sephadex LH-20 column (1.2 × 60 cm) eluted with 120 mL MeOH to give six fractions (Fr.1~6), which were analyzed by the GC/MS method and gave six total ion chromatograms (Figure 3). From these ion chromatograms, we found that 2-(2-phenylethyl)chromones were concentrated in Fr. 3 and Fr. 4, and especially Fr.4 possessed the best resolution ratio. Thus the compounds of Fr. 4 were further characterized by NIST05 and WILEY275 database matching, and then the MS spectra of unidentified compounds were drawn and characterized by the methods and rules mentioned above, and referring to the MS data in Table 1. Finally fifteen compounds were characterized as 2-(2-phenylethyl)chromones, one of them from Fr. 3 (Fr. 3*-*23). The results are shown in Table 3, and Figure 4. The detailed identification procedures are as follows: first, the number of hydroxy and methoxy groups can be deduced on the basis of the molecular ion peak or molecular weight (MW) through the formula (30m + 16n = MW − 250) in Table 3. Then we found that all the base peak of compounds Fr. 4-10~Fr. 4-26 are benzyl fragment peaks (*m/z* 91, 121, 137), which are generated by the first cleavage method and in accordance with the conditions of rules 1(a)~1(c), and 2(b).

**Table 3:** 2-(2-Phenylethyl)chromone preliminarily identified by their MS characterization and chemical components identified by database matching of Fraction 4 of agarwood oil.

No.	Retention time	Compounds identified by database matching	Molecular formula				Molecular weight	
Fr.4-1	6.21	Benzenepropanoic acid	C_9_H_10_O_2_				150	
Fr.4-2	6.64	Melitol	C_9_H_8_O_2_				148	
Fr.4-3	7.22	3,4-dimethoxy-Phenol	C_8_H_10_O_3_				154	
Fr.4-4	7.90	Anisylacetone	C_11_H_14_O_2_				178	
Fr.4-5	8.72	Raspberry ketone	C_10_H_12_O_2_				164	
Fr.4-6	9.13	3-(4-Methoxyphenyl)propionic acid	C_11_H_16_O_2_				180	
Fr.4-7	10.01	Zingerone	C_11_H_14_O_3_				194	
Fr.4-8	11.19	Methylm-methoxymandelate	C_10_H_12_O_4_				196	
Fr.4-9	16.47	1,5-diphenyl-1-Penten-3-one	C_17_H_16_O				236	
**No.**	**Retention time**	**2-(2-Phenylethyl)chromones preliminarily identified by their MS characterization**	**Substituent condition**				**MS characterization**	
			**Chromone moiety**		**Benzyl moiety**		**Molecular ion peak**	**Base peak**
			**OH**	**OCH_3_**	**OH**	**OCH_3_**		
Fr.4-10	19.04	2-(2-phenylethyl)chromone	0	0	0	0	250	91
Fr.4-11	22.33	No.2 or No.3 or No.4	1	0	0	0	266	91
Fr.4-12	22.88	No. 8	0	1	0	0	280	91
Fr.4-13	23.63	Similar compound as No. 14	1	1	0	0	296	91
Fr.4-14	23.90	No. 10 or No. 11	2	0	0	0	282	91
Fr.4-15	25.24	No.2 or No.3 or No.4	1	0	0	0	266	91
Fr.4-16	25.97	Similar compound as No. 14	1	1	0	0	296	91
Fr.4-17	26.14	Two compounds mixture					296,310	91,121
Fr.4-18	26.81	No. 19	0	2	0	0	310	91
Fr.4-19	27.39	No.22 or No. 23	2	0	0	1	312	121
Fr.4-20	27.92	Similar compound as No. 14	1	1	0	0	296	91
Fr.4-21	28.67	No.15	1	0	0	1	296	121
Fr.4-22	29.52	Similar compound as No. 38	1	1	0	1	326	121
Fr.3-23	30.02	Similar compound as No. 39	2	0	1	1	328	137
Fr.4-24	30.12	New compound	3	0	0	1	328	121
Fr.4-25	30.15	No.29	0	2	0	1	340	121
Fr.4-26	31.25	Similar compound as No. 38	1	1	0	1	326	121

All the base peaks of Fr.4-10~Fr.4-16, Fr.4-18 and Fr.4-20 are *m*/*z* 91, which indicates these molecules have a non-substituted benzyl moiety and the substituent groups are located on the chromone moiety. The molecular ion peak of Fr.4-11 and Fr.4-15 is *m*/*z* 266, which indicates there is only one hydroxy substituent group in each of them. Since the benzyl moiety is non-substituted, the hydroxy is substituted on the chromone moiety. The position of substitution is difficult to determine, so their structures should be one of No. 2~No. 4, respectively. Similarly, the molecular ion peak of Fr.4-14 is at *m*/*z* 282, which indicates the chromone moiety is substituted by two hydroxyl groups, so its structure may be No. 10 or No. 11. The mass spectra of Fr.4-13, Fr.4-16, Fr.4-20 are quite similar, with the same molecular ion peak at *m*/*z* 296, which indicates the chromone moieties are substituted by one hydroxy and one methoxy, similar to No. 14. Finally, only compound No. 8 possess similar MS data as that of Fr.4-12, therefore Fr.4-12 is identified as No. 8. Similarly, Fr.4-18 is identified as No. 19.

All the base peaks of Fr.4-19, Fr.4-21, Fr.4-22, Fr.4-24, Fr.4-25 and Fr.4-26 are *m*/*z* 121, which indicates these molecules have a methoxy-substituted benzyl moiety, and the position of the methoxy group is most likely at the *p-*position (position 4′), while the other substituent groups are located on the chromone moiety. The molecular ion peak of Fr.4-19 is at *m*/*z* 312, indicating the chromone moiety is substituted by two hydroxy groups, so its structure is likely to be No. 22 or No. 23. The mass spectra of Fr.4-22 and Fr.4-26 are very similar, with the same molecular ion peak at *m*/*z* 326, which indicates that their chromone moieties are substituted by one hydroxy and one methoxy, similar to No. 38. Only compound No. 15 possesses similar MS data to that of Fr.4-21, therefore Fr.4-21 is identified as No.15. Similarly, Fr.4-25 is identified as No. 29. The molecular ion peak of Fr.4-24 is at *m*/*z* 328, which indicates the chromone moiety is substituted by three hydroxy groups. Such a compound has not been reported, so Fr.4-24 should be a new compound.

The base peak of Fr.3-23 is *m*/*z* 137, which indicates the molecule has a *p*-methoxy- and a hydroxyl-substituted benzyl moiety. The molecular ion peak is at *m*/*z* 328, which indicates the chromone moiety is substituted by two hydroxy groups, so its structure is likely to be No. 39.

There are two molecular ions at *m*/*z* 296 and *m*/*z* 310, and two fragment peaks at *m*/*z* 121 (100%) and *m*/*z* 91 (94%) in the mass spectrum of Fr.4-17, indicating a mixture of two compounds.

2-(2-Phenylethyl)chromone derivatives, mainly found in agarwoods from the *Aquilaria* species [18,19,21,22,23,24,25,26,27,28,29,30,31], and rarely found in other species [20,32,33,34], are irreplaceable components for the special fragrance of agarwood, but they have seldom been characterized by GC-MS [14,15,16,17], which was often used to analyze the constituents of volatile oil of agarwood and many sesquiterpenes, such as agarospirol, baimuxinal, baimuxinol, together with some simple volatile aromatic compounds, such as benzylacetone, anisylacetone, have been characterized [35,36,37]. One reason maybe there are not too many MS spectra of 2-(2-phenylethyl)chromones in the database for matching, and people know little about the MS characterization of 2-(2-phenylethyl)chromones. The melting points of most of 2-(2-phenylethyl)chromones are under 200 °C, which are suitable for GC-MS analysis. Future work will focus on obtaining more 2-(2-phenylethyl)chromones standards to enrich the MS spectra database, so that a more comprehensive characterization of these compounds by GC-MS analysis is possible.

## 3. Experimental

### 3.1. GC-MS Analysis

A Hewlett Packard GC 6890 gas chromatography instrument coupled with a Mass Selective Detector (5975C, Agilent Technologies, Santa clara, CA, USA) was used for the analysis. Separation of the samples by gas chromatography was carried out using a Zebron ZB-5MSi 5% Phenyl 95% Dimethylpolysiloxane capillary column (30 m × 0.25 mm × 0.25 µm) (Phenomenex, Torrance, CA, USA). Samples (0.5 mg) were dissolved in dichloromethane, then 1.0 µL of solvent was injected into the front inlet of the gas chromatograph operating at 250 °C with 40:1 of split ratio. The flow rate of the helium (carrier gas) was 1.0 mL/min. For the agarwood oil, the oven program commenced at 50 °C and increased to 250 °C at a rate of 5 °C/min, and finally increased to 310 °C at a rate of 10 °C/min, then held for 10 min. For Fr.1~6, the oven program commenced at 100 °C and increased to 200 °C at a rate of 10 °C/min, and finally increased to 310 °C at a rate of 5 °C/min, then held for 10 min. The interface temperature was 280 °C. Ionization of the compounds by electron impact (EI) was obtained using an emission current of 70 eV. The ion source temperature was set at 230 °C and scan scope was from 20 to 450 amu. Figure 2 and Figure 3 show the total ion chromatograms of the agarwood oil and Fr.1~6. The relative contents of the compounds were determined by normalization. The compounds were characterized by NIST05 and WILEY275 database matching.

### 3.2. Plant Material

**Figure 5:**
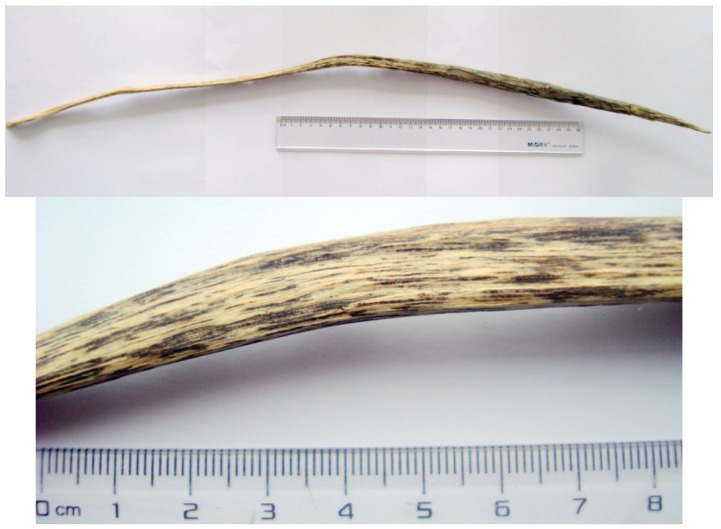
The photo of the artificial agarwood sample. (**a**) The whole piece of the agarwood. (**b**) The partially magnified agarwood.

The agarwood inducer, which remains a technical secret, was injected into the trunk of six-year-old *A. sinensis* trees on an *A. sinensis* plantation located at Yanfeng Town at Haikou City, Hainan Province, China, at September, 2012. The agarwood was then harvested at May, 2013, about nine months after agarwood-inducing operation. The dark resins of the agarwood were exposed after getting rid of white wood (Figure 5). On the surface of this agarwood, the lines of dark resins could be clearly seen by using magnifying glass, and the pleasant fragrance could be smelled after burning. The specimen (YF 20130502) is deposited at Institute of Tropical Bioscience and Biotechnology, Chinese Academy of Tropical Agricultural Sciences.

### 3.3. Extraction and Separation

The crushed agarwood (1.32 g, dry weight) was soaked in Et_2_O (3 × 10 mL) and extracted by the ultrasound method. The Et_2_O extract (30 mL) was filtered and evaporated to get brownish yellow oil (74.4 mg, 5.6% yield). A sample of this oil (0.5 mg) was analyzed by GC-MS. Another sample of oil (70 mg) was dissolved in 0.5 mL MeOH, and then applied to a Sephadex LH-20 column (1.2 × 60 cm) eluted with 120 mL MeOH to separate it into six fractions (Fr.1~6).

## 4. Conclusions

MS characterization of all the 2-(2-phenylethyl)chromones in agarwood was summarized in Table 1 and a formula (30m + 16n = MW − 250) was provided to calculate the number of methoxy (m) or hydroxy (n) groups according to the molecular ion peak or molecular weight (MW) of 2-(2-phenylethyl)chromones only substituted by methoxy or/and hydroxy groups. We deduced that the characteristic fragmentation behavior of the 2-(2-phenylethyl)chromones is the cleavage of the CH_2_-CH_2_ bond between chromone moiety and phenyl moiety. Thus, characteristic fragment ions, such as *m*/*z* 91 [C_7_H_7_], 107 [C_7_H_6_+OH], 121 [C_7_H_6_+OCH_3_], 137 [C_7_H_5_+OH+OCH_3_] are formed by differently substituted benzyl moieties, while characteristic fragment ions, such as *m*/*z* 160 [C_10_H_8_O_2_], 176 [C_10_H_7_O_2_+OH], 190 [C_10_H_7_O_2_+OCH_3_], 220 [C_10_H_6_O_2_+OCH_3_×2] are formed by differently substituted chromone moieties. Furthermore, rules regarding to the relationships between the positions of the hydroxy or methoxy groups and the relative abundances of benzyl and chromone fragment ions have been determined. Elucidation of how the positions of hydroxy or methoxy groups affect the relative abundances of benzyl and chromone fragment peaks are also provided. In conclusion, 2-(2-phenylethyl)chromones can be preliminarily determined by their MS spectra. The above mentioned methods and the MS data in Table 1 were applied to the analysis in an agarwood sample. Fifteen unidentified compounds analyzed by GC-MS and NIST05 and WILEY275 database matching were preliminarily determined as 2-(2-phenylethyl)chromones by analysis of their MS spectra and by comparison of their spectra with those in Table 1, which give a good example and proved the methods are feasible. The methods presented in this paper should be helpful for the analysis and characterization of 2-(2-phenylethyl)chromones in agarwood by GC-MS, and provides a scientifically sound, fast and reliable evaluation method for the quality of agarwood.
